# A comparative evaluation of thiol/disulfide homeostasis and ischemia-modified albumin levels in patients with heroin use disorder in remission and detoxification

**DOI:** 10.3389/fnbeh.2026.1933257

**Published:** 2026-10-02

**Authors:** Şafak Yalçin Şahiner, Dilek Baysal, Tuğberk Vayisoğlu, Zehra Uçar Hasanli, Salim Neşelioğlu, Gamze Avcioğlu, Hakkı Polat, Rifat Serav İlhan, Özcan Erel

**Affiliations:** 1 Faculty of Medicine, Department of Psychiatry, Ankara University, Ankara, Türkiye; 2 Combating for Addiction Application and Research Center, Ankara University, Ankara, Türkiye; 3 Faculty of Medicine, Department of Psychiatry, Hacettepe University, Ankara, Türkiye; 4 Alcohol and Drug Addiction Treatment Center, Ankara Training and Research Hospital, Ankara, Türkiye; 5 Faculty of Medicine, Department of Clinical Biochemistry, Yıldırım Beyazıt University, Ankara, Türkiye; 6 Department of Medical Biochemistry, Karadeniz Ereğli State Hospital, Zonguldak, Türkiye; 7 Institutional Big Data Management Center, Middle East Technical University, Ankara, Türkiye

**Keywords:** detoxification, heroin use disorder, ischemia-modified albumin, redox, remission, thiol-disulfide balance

## Abstract

**Introduction:**

Chronic heroin use disturbs body’s redox (oxidant-antioxidant) balance, together with dynamic thiol-disulfide balance and ischemia-modified albumin; which are two distinct blood markers that may reflect the oxidative and ischemic load in substance dependence. It is still unclear whether these markers change across the clinical phases of heroin use disorder (HUD), or whether they relate to the affective and behavioral symptoms that dominate the clinical picture.

**Methods:**

In this cross-sectional study, 101 adults meeting DSM-5 criteria for HUD were split into a remission group (*n* = 52) and a detoxification group (*n* = 49). We measured serum native and total thiol, disulfide, the disulfide/native thiol, disulfide/total thiol and native thiol/total thiol ratios, and IMA. Participants also completed a symptom inventory, a craving measure, and scales for healthy-lifestyle behavior and nutrition attitude. Groups were compared with Student’s *t*-test or the Mann–Whitney *U* test, categorical variables with chi-square or Fisher’s exact test; associations were tested with Spearman correlations, and predictors of total thiol and IMA with multiple linear regression.

**Results:**

Patients in detoxification group scored markedly higher on depression, anxiety, anger, somatic symptoms, suicidal ideation, psychosis, sleep disturbance, dissociation and craving (all *p* ≤ 0.001), and lower on healthy-lifestyle and nutrition-attitude scores (*p* < 0.001). In contrast, no thiol/disulfide measure or IMA differed between phases. IMA correlated positively with depression (*r* = 0.254) and craving (*r* = 0.209); whereas the disulfide/thiol ratios correlated negatively, and native thiol/total thiol positively with healthy-lifestyle and nutrition-attitude scores. Regression models for total thiol and IMA were not statistically significant.

**Conclusion:**

In HUD, redox and ischemia markers appear to follow symptom severity; especially depression, craving and health behavior, rather than the treatment phase itself. IMA, in particular, may reflect an oxidative and inflammatory component of mood regulation and craving.

## 1 Introduction

Heroin use disorder (HUD) is one of the most disabling forms of substance dependence, carrying a heavy burden of psychiatric comorbidity, social impairment and premature mortality (Pathan and Williams, 2012; Baldo, 2021; Milella et al., 2023). Although opioids exert their reinforcing and withdrawal effects largely through well-defined receptor systems, repeated heroin exposure also reshapes cellular physiology well beyond classical neurotransmission systems (Nestler, 2001). A recurring theme in this area is a disruption in the balance between pro-oxidant and antioxidant processes, a state referred to as oxidative stress (Viola et al., 2023).

Aerobic metabolism unavoidably generates reactive oxygen species, which cells counteract through antioxidant defenses. When this balance shifts toward oxidation, lipids, proteins, and nucleic acids sustain oxidative damage (Jomova et al., 2023). Thiol groups, carried on cysteine residues, play a central role in maintaining redox balance. Under oxidizing conditions, thiol (-SH) groups form reversible disulfide bonds (-S-S-), and the dynamic equilibrium between these groups, known as thiol-disulfide homeostasis, provides insight into the body’s redox state (Erel and Neselioglu, 2014; Erel and Erdoğan, 2020). Since the majority of plasma thiols reside on human serum albumin, this protein serves both as the largest reservoir of circulating antioxidant capacity and as a convenient marker for assessing systemic redox status (Bocedi et al., 2018; Turell et al., 2013).

The same albumin molecule provides a second, complementary marker. The N-terminus of the molecule binds transition metals, but under hypoxic and acidic conditions, this region undergoes structural modification and loses its binding affinity, resulting in ischemia-modified albumin (IMA) (Shevtsova et al., 2021). Originally described in the context of cardiac ischemia, IMA has more recently been recognized as a broader indicator of combined oxidative stress, ischemia, and inflammation, with elevated levels reported in several psychiatric conditions, including major depressive disorder and obsessive-compulsive disorder (Karaaslan et al., 2019; Tunç et al., 2019; Tampa et al., 2022).

In addictions specifically, altered thiol-disulfide homeostasis and oxidative enzyme activity have been reported in heroin, methamphetamine and mixed substance use (Soykut et al., 2013; Najafi et al., 2017; Kotan et al., 2017; Turan et al., 2023; Kabadayı Şahin et al., 2023). Chronic opioid use is also accompanied by systemic immune responses and altered inflammatory markers (Kholodnaia et al., 2022; Wang et al., 2023; Baykara et al., 2021), and neuroimmune processes have been associated with both the maintenance of and recovery from opioid use disorder (Butelman et al., 2023). However, the direction of these redox changes has not been consistent across studies, suggesting that the markers may depend as much on a patient’s clinical state and treatment phase as on the substance itself (Ergin Tuncay et al., 2023; Rowland et al., 2018). This is clinically relevant, as HUD is not a static condition: patients transition between acute detoxification, marked by intense affective and craving symptoms, and longer periods of remission, often under maintenance pharmacotherapy. A companion case–control study from our group reported differences in disulfide-related ratios and IMA between patients with HUD and healthy controls (Yalcin Sahiner et al., 2025). However, whether these markers also distinguish between phases of the disorder, or instead relate to the affective and behavioral symptoms that vary within patients, has yet to be examined.

Emotional dysregulation, depressed mood, anxiety, and craving are common and frequently fluctuating features of opioid dependence, most of which are linked to oxidative and inflammatory signaling (Sloan et al., 2017; Jiang et al., 2022). If peripheral redox and ischemia markers reflect this symptom burden, they may serve as dimensional indicators of illness severity rather than merely as categorical markers that assign patients into groups. Building on this reasoning, this study had three aims: (i) to compare thiol-disulfide parameters and IMA between patients in remission and patients undergoing detoxification; (ii) to characterize the psychiatric, substance-related behavioral, and health-behavior profiles of the two groups; and (iii) to test the association of redox/ischemia markers with symptom severity, substance-related problems, and health behaviors, as well as the prediction of total thiol and IMA by clinical variables. Moreover, rather than evaluating biological markers in clinical isolation, the primary aim of our cross-sectional study was to examine systemic oxidative stress (thiol/disulfide balance) and ischemia (IMA) within a holistic biopsychosocial framework in patients with heroin use disorder. Specifically, we aimed to investigate whether these peripheral redox and ischemia markers differ between distinct clinical treatment stages (acute detoxification versus sustained remission) and how they interact with psychiatric symptom burden, substance craving severity, and health-protective behaviors (nutritional attitudes and healthy lifestyle practices). By simultaneously analyzing these psychometric, behavioral, and biochemical dimensions across the two clinical groups, the study aims to clarify whether peripheral biomarkers primarily reflect categorical treatment status or instead track the dimensional severity of psychological distress and lifestyle factors.

## 2 Materials and methods

### 2.1 Study design and participants

This single-center study is a cross-sectional, two-group design. All participants were adults aged 18–65 years with a DSM-5 diagnosis of opioid use disorder, established by the attending physicians of the Alcohol and Substance Treatment Center and verified through hospital and patient records. Patients were assigned to one of two groups according to their clinical phase at the time of assessment. Each patient was classified by treatment phase at the time of assessment: patients in stable and sustained abstinence under routine outpatient follow-up formed the remission group, while those undergoing active management of acute withdrawal formed the detoxification group.

Patients were enrolled in the study between September 2025 and December 2025. Of the records screened, one was excluded because it contained no usable data. The final sample comprised 101 patients: 52 in the remission group and 49 in the detoxification group. Both men and women were eligible for inclusion. Each participant attended a single assessment session, during which the self-report scales were completed and a venous blood sample was collected for biochemical analysis.

The study was conducted in accordance with the Declaration of Helsinki and Good Clinical Practice standards, and was approved by the Ankara University Ethics Committee (Date: 20 August 2025, No.: 2025/498). After a full explanation of the study procedures, all participants provided written informed consent.

Inclusion criteria for both groups:

- A DSM-5 diagnosis of heroin use disorder
- Age between 18 and 65 years
- Sufficient fluency in Turkish to complete the assessment
- Ability to read and write
- No psychiatric diagnosis other than heroin use disorder
- Absence of any medical or neurological disorders

Group assignments made by treatment phases:

- Remission group: Sustained abstinence with stabilized outpatient follow-up for at least 3 months
- Detoxification group: acute phase of withdrawal management

Exclusion criteria (both groups):

- Age outside the 18–65 range
- Insufficient proficiency of Turkish, or inability to read and write
- An unstable or acute medical or neurological condition
- A psychiatric diagnosis other than heroin use disorder, or use of substances other than heroin
- Unwillingness to provide informed consent

### 2.2 Clinical aspects and psychometric assessment

All participants completed the sociodemographic and clinical information form, the DSM-5-TR Self-Rated Level 1 Cross-Cutting Symptom Measure (Adults), the Penn Alcohol Craving Scale, the Attitude Scale for Healthy Nutrition, and the Healthy Lifestyle Behavior Scale themselves during a single interview. The sociodemographic and clinical information form was developed by the researchers and serves to collect not only the patients’ sociodemographic details but also information regarding their treatment and history of heroin use.

DSM-5-TR Self-Rated Level 1 Cross-cutting Symptom Measure (Adults): This measure assists researchers and clinicians in assessing participants’ symptoms and their severity across various areas of mental health. It comprises 13 sub-domains, including depression, anger, mania, anxiety, somatic symptoms, suicidal ideation, psychosis, sleep problems, memory, repetitive thoughts and behaviors, dissociation, personality, and substance use, and consists of 23 questions. Each section contains a different number of questions and is administered as a self-report scale. This scale functions as a broad self-report screening instrument covering various psychiatric domains, rather than as a diagnostic clinical instrument. The findings obtained from this scale reflect self-reported symptom scores and screening levels, rather than definitive clinical diagnoses. The DSM-5-TR Self-Rated Level 1 Cross-Cutting Symptom Measure uses a 5-point Likert-type scale (0 = none, 1 = mild—1 or fewer days, 2 = moderate—a few days, 3 = moderate—on more than half the days, and 4 = severe—almost every day). After the participant completes the measure, the administrator reviews each score marked for every item and determines the highest score obtained from any item within a section. The validity and reliability study of the Turkish adult version of the measure was conducted by Çökmüş et al. (2017). According to this study, the scale’s internal consistency was found to be 0.94, and the item-total correlation coefficients ranged from 0.30 to 0.79 (Çökmüş et al., 2017).

Substance Craving Scale: Heroin craving was assessed using the Turkish adaptation of the original Penn Alcohol Craving Scale (PACS) developed by Flannery et al. (1999), in which the term “alcohol” was replaced with “substance.” This adaptation was carried out by Evren et al. (2011), who named it the Substance Craving Scale (Madde Aşerme Ölçeği-MAO). The Turkish version of the scale has been shown to be a valid and reliable instrument for assessing craving in substance-dependent populations (Evren et al., 2011). Participants included in our study were asked to consider their heroin use and craving specifically over the past week and to complete this 5-item self-report scale accordingly.

The Attitude Scale for Healthy Nutrition (ASHN): A psychometric measure developed to assess individuals’ attitudes toward healthy eating. It was developed by Tekkurşun-Demir and Cicioğlu in 2019. The scale consists of 21 items grouped under four factors: Information on Nutrition (IN), Emotion for Nutrition (EN), Positive Nutrition (PN), and Malnutrition (MP). Using a 5-point Likert-type rating scale, it aims to measure individuals’ beliefs, attitudes, and tendencies regarding healthy eating. Reliability analyses, including the internal consistency coefficient and test–retest method, have established that the scale is a reliable measurement tool. Item analysis results have demonstrated that the scale items are discriminant (Tekkurşun-Demir and Cicioğlu, 2019).

The Healthy Lifestyle Behavior Scale: A valid and reliable self-report instrument developed to assess individuals’ healthy lifestyle habits in a holistic manner. Developed by Sayili and colleagues in 2024, the scale uses a 5-point Likert-type rating system. The total score is calculated on a scale of 0–100 by multiplying the average of all items by 25. Internal consistency reliability was assessed using Cronbach’s alpha coefficient and was found to be 0.863 for the scale as a whole; for the sub-dimensions, these values are above 0.70 (Sayili et al., 2024).

In both groups, heroin use disorder was diagnosed by the physicians of the unit in accordance with DSM-5 criteria. In line with the study protocol, no restriction was placed on the type of clinical admission. Suitability was first checked against hospital records and then confirmed by reviewing individual patient files, so that every enrolled patient met the predefined criteria. The center where the study was conducted provides dedicated outpatient and inpatient care for alcohol and substance use disorders, which made it possible to sample both treatment phases within a single setting.

Patients who met the inclusion criteria were enrolled on the same day. After providing written informed consent, each participant was seen in a designated interview room, where the clinical and psychometric assessments were completed in a single session lasting approximately 30 min. A venous blood sample was then collected in the phlebotomy unit for biochemical analysis. The same procedure was followed for patients in remission and patients in detoxification, and the assessments for the two groups were carried out over the same period, thereby maintaining methodological consistency throughout the study.

### 2.3 Blood sampling and biochemical analysis

Blood samples were obtained from the addicts in plain tubes. Samples were left to stand at room temperature for 30 min and then centrifuged at 3500 rpm for 10 min. The serum was separated from the samples, transferred to Eppendorf tubes, and stored at −80 °C until analysis.

#### 2.3.1 Thiol-disulfide homeostasis

Thiol-disulfide parameters were measured using the automated spectrophotometric assay of Erel and Neselioglu (2014). Dynamic disulfide bonds were first reduced to free thiol groups with sodium borohydride, and the unreacted borohydride was then quenched with formaldehyde to prevent it from interfering with the subsequent reaction with 5,5′-dithiobis-(2-nitrobenzoic acid) (DTNB). Following the DTNB reaction, native and total thiol concentrations were determined, and the disulfide content was calculated as half the difference between total and native thiol. From these measurements, the disulfide/native thiol, disulfide/total thiol, and native thiol/total thiol ratios, together with the disulfide amount, were calculated.

#### 2.3.2 Ischemia-modified albumin (IMA)

IMA was quantified with the albumin-cobalt binding (ACB) test (Bar-Or et al., 2001). Briefly, 50 μL of 0.1% cobalt(II) chloride (CoCl₂·6H₂O; Sigma-Aldrich Chemie GmbH, Steinheim, Germany) was added to serum and mixed; 50 μL of dithiothreitol (1.5 mg/mL) was then added, followed by a 10-min incubation. The reaction was stopped approximately 2 min later with 1.0 mL of 0.9% sodium chloride. A blank was prepared in parallel by substituting distilled water for dithiothreitol. Absorbance was measured at 480 nm on a spectrophotometer, and results were expressed in absorbance units (ABSU) (Eroglu et al., 2023).

### 2.4 Statistical analysis

Statistical analyses were conducted using IBM SPSS Statistics 23. Normally distributed continuous variables were summarised as mean ± standard deviation, non-normally distributed continuous variables as median (minimum-maximum), and categorical variables as percentage (*n*). Between-group comparisons were performed using Student’s *t*-test for normally distributed continuous variables and the Mann–Whitney *U* test for non-normally distributed continuous variables. Categorical variables were compared using Pearson’s chi-square test or Fisher’s exact test, as appropriate. Associations between variables were evaluated using Spearman’s rank correlation coefficient.

The determinants of total thiol and ischemia-modified albumin (IMA) were examined using hierarchical multiple linear regression. In Model 1, age, body mass index (BMI), alcohol use, duration of heroin use, daily heroin amount, medication use, and time since last heroin use were entered as predictors. Alcohol use was dichotomized as no current alcohol use versus any current alcohol use for the regression analyses. In Model 2, depression, substance craving, and healthy lifestyle behavior scores were additionally entered to evaluate their incremental contribution beyond the baseline clinical and substance-use variables. Changes in explained variance (Δ*R*^2^) were examined between models. Multicollinearity was assessed using tolerance and variance inflation factor (VIF) statistics.

Because medication use differed markedly between the remission and detoxification groups, additional medication-adjusted analyses of the redox and ischemia biomarkers were performed using general linear models including treatment group and medication use as main effects. Estimated marginal means were obtained for treatment-group comparisons. The group-by-medication interaction was not included because of the highly unbalanced distribution of medication use across treatment groups. Statistical significance was set at *p* < 0.05 In addition to *p*-values, effect sizes were calculated for group comparisons (Cohen’s *d* for *t*-tests and the rank-biserial correlation for Mann–Whitney *U* tests), together with 95% confidence intervals (CI). Because a large number of correlations were tested (Table 1), a Bonferroni-corrected threshold (*α* = 0.05/35 = 0.0014) was additionally applied to those analyses. To address potential confounding, we repeated the treatment-phase comparisons of IMA and total thiol in multivariable linear models adjusting for medication use, recent non-heroin substance use, duration of heroin use and age at first treatment, and in a sensitivity analysis restricted to patients without recent non-heroin substance use. Post-hoc statistical power for the IMA comparison was estimated for the observed effect size.

**Table 1:** Spearman correlations between biomarkers and psychometric measures.

Variable	Depression	Anxiety	Substance craving (total)	Healthy lifestyle behavior (total)	Healthy nutrition attitude (total)
Native thiol (μmol/L)	−0.024	−0.124	−0.124	0.118	0.058
Total thiol (μmol/L)	0.010	−0.103	−0.106	0.073	−0.008
Disulfide (μmol/L)	0.135	0.044	0.048	−0.168	−0.142
Disulfide/Native thiol	0.158	0.126	0.121	−0.237^*^	−0.203^*^
Disulfide/Total thiol	0.158	0.126	0.121	−0.237^*^	−0.203^*^
Native thiol/Total thiol	−0.158	−0.126	−0.121	0.237^*^	0.203^*^
IMA (ABSU)	0.254^*^	0.011	0.209^*^	−0.077	−0.070

Values are Spearman’s rank correlation coefficients (*r*). *p < 0.05 (two-tailed). No correlation remained significant after Bonferroni correction (*α* = 0.05/35= 0.0014).

## 3 Results

### 3.1 Sample characteristics

Table 2 shows sociodemographic characteristics of two groups and their comparison. The sample was predominantly male (95/101, 94.1%), with a mean age of 33.0 ± 6.3 years and a mean body mass index of 23.8 ± 4.2 kg/m^2^. Most patients were single (55.4%) or married (34.7%), had completed 9–12 years (48.5%) or 6–8 years (28.7%) of education, were employed (69.3%), and lived with their parents (55.4%) or with a spouse (29.7%); reported income was above the minimum wage in about half (49.5%). The remission and detoxification groups did not differ significantly on any sociodemographic variable, including age, body mass index, sex, marital status, education, employment, income, co-residing persons, alcohol use, or family history of substance use (all *p* > 0.05).

**Table 2:** Comparative evaluation of sociodemographic data in the remission and detoxification groups.

Variable	Remission (*N* = 52)	Detoxification (*N* = 49)	Test	*p*
Age (years)	33.50 (23–59)	31.00 (20–49)	MWU = −1.58	0.115
BMI (kg/m^2^)	24.60 ± 5.03	23.02 ± 2.90	*t* = 1.91	0.059
Sex			Fisher	1.000
Female	5.8 (3)	6.1 (3)		
Male	94.2 (49)	93.9 (46)		
Marital status			*χ*^2^ = 1.31, df = 2	0.519
Married	38.5 (20)	30.6 (15)		
Single	50.0 (26)	61.2 (30)		
Divorced	11.5 (6)	8.2 (4)		
Education level			*χ*^2^ = 0.83, df = 3	0.841
1–5 years	1.9 (1)	2.0 (1)		
6–8 years	25.0 (13)	32.7 (16)		
9–12 years	50.0 (26)	46.9 (23)		
≥13 years	23.1 (12)	18.4 (9)		
Employment status			*χ*^2^ = 4.77, df = 2	0.092
No	19.2 (10)	38.8 (19)		
Yes	78.8 (41)	59.2 (29)		
Retired	1.9 (1)	2.0 (1)		
Income level			*χ*^2^ = 1.06, df = 2	0.588
Below minimum wage	17.3 (9)	24.5 (12)		
Minimum wage	28.8 (15)	30.6 (15)		
Above minimum wage	53.8 (28)	44.9 (22)		
Co-residing persons			*χ*^2^ = 3.00, df = 3	0.392
Parents	50.0 (26)	61.2 (30)		
Spouse	36.5 (19)	22.4 (11)		
Child	3.8 (2)	2.0 (1)		
Other	9.6 (5)	14.3 (7)		
Alcohol use			*χ*^2^ = 4.81, df = 4	0.307
None	61.5 (32)	55.1 (27)		
Monthly	34.6 (18)	30.6 (15)		
Weekly	3.8 (2)	6.1 (3)		
3–4/week	0.0 (0)	4.1 (2)		
Daily	0.0 (0)	4.1 (2)		
Family history of substance use			Fisher	0.111
No	100.0 (52)	93.9 (46)		
Yes	0.0 (0)	6.1 (3)		
Smoking amount (packs/day)			*χ*^2^ = 1.46, df = 4	0.834
Non-smoker	2.0 (1)	2.0 (1)		
<1 pack/day	23.5 (12)	18.4 (9)		
1 pack/day	56.9 (29)	57.1 (28)		
2 packs/day	17.6 (9)	20.4 (10)		
≥3 packs/day	0.0 (0)	2.0 (1)		

Continuous variables are mean ± SD; categorical variables are % (*n*). *t*, Student’s *t*-test; MWU, Mann–Whitney *U* test; *χ*^2^, Pearson chi-square; Fisher, Fisher’s exact test; df, degrees of freedom; BMI, body mass index. *p* < 0.05 statistically significant.

Clinical and substance-use characteristics are summarised in Table 3. Across the whole sample, the mean duration of heroin use was 9.18 ± 4.89 years (range 1–31 years), and the longest continuous period of use averaged 6.79 ± 3.97 years (range 1–21 years); patients used a mean of 3.11 ± 1.82 g of heroin per day (range 1–10), and the mean age at first treatment was 24.72 ± 6.54 years (range 12–45). Foil/inhalation was the most common route of use (83 patients, 82.2%), followed by combined injection and foil use (15, 14.9%) and injection alone (3, 3.0%). Twenty-six patients (25.7%) reported recent use of a substance other than heroin, 12 (11.9%) had a chronic medical condition, and almost all were current smokers, with only two reporting that they did not smoke. At assessment, 67 patients (66.3%) were receiving pharmacotherapy, predominantly buprenorphine/naloxone (62 patients, 61.4%).

**Table 3:** Clinical and substance-use characteristics of the remission and detoxification groups.

Variable	Remission (*N* = 52)	Detoxification (*N* = 49)	Test	*p*
Duration of heroin use (years)	8.00 (2–16)	10.00 (1–31)	MWU *z* = −2.58	0.010
Longest continuous use (years)	5.00 (2–17)	8.00 (1–21)	MWU *z* = −2.30	0.022
Daily heroin amount (g)	2.75 (1–10)	2.00 (1–10)	MWU *z* = −1.25	0.213
Age at first treatment (years)	26.17 ± 6.74	23.18 ± 6.02	*t* = 2.35	0.021
Medication use (yes)	98.1 (51)	32.7 (16)	*χ*^2^ = 48.35	<0.001
Buprenorphine/naloxone use	94.1 (48/51)	87.5 (14/16)	Fisher	0.586
Medication type: Suboxone†	96.0 (48)	100.0 (14)	Fisher	1.000
Regular medication use (yes)‡	98.0 (50/51)	45.8 (22/48)	–	–
Regular medication-use duration			–	–
Active	0.0 (0)	100.0 (49)		
0–6 months	7.7 (4)	0.0 (0)		
6 months-1 year	11.5 (6)	0.0 (0)		
>1 year	80.8 (42)	0.0 (0)		
Non-heroin substance use (yes)	9.6 (5)	42.9 (21)	*χ*^2^ = 14.58	<0.001
Family history of substance use (yes)	0.0 (0)	6.1 (3)	Fisher	0.111
Chronic medical condition (yes)	15.7 (8)	8.2 (4)	*χ*^2^ = 1.34	0.247
Current smoker (yes)	98.0 (50)	98.0 (48)	Fisher	0.977
Route of use			*χ*^2^ = 2.89	0.236
Injection	1.9 (1)	4.1 (2)		
Foil/inhalation	88.5 (46)	75.5 (37)		
Injection + foil	9.6 (5)	20.4 (10)		

Continuous variables are mean ± SD; categorical variables are % (*n*). *t*, Student’s *t*-test; MWU, Mann–Whitney *U* test; *χ*^2^, Pearson chi-square; Fisher, Fisher’s exact test. *p* < 0.05 statistically significant. Percentages for specific medication types (Buprenorphine/naloxone) were calculated using the number of patients receiving active pharmacological treatment in each group as the denominator (Remission: *n* = 51; Detoxification: *n* = 16).

Several clinical characteristics differed by treatment phase (Table 3). Patients in the detoxification group had a longer duration of heroin use [median 10.00 (1–31) vs. 8.00 (2–16) years; *p* = 0.010] and a longer period of continuous use [8.00 (1–21) vs. 5.00 (2–17) years; *p* = 0.022], and they had entered treatment at a younger age (23.18 ± 6.02 vs. 26.17 ± 6.74 years; *p* = 0.021). Medication use was substantially more frequent in the remission group (51/52, 98.1%) than in the detoxification group (16/49, 32.7%; *p* < 0.001). Among participants with available medication-name data, Suboxone was the predominant pharmacotherapy. Recent use of a non-heroin substance was more common in the detoxification group (21/49, 42.9% vs. 5/52, 9.6%; *p* < 0.001). Daily heroin amount, route of use, family history of substance use, chronic illness and smoking status did not differ significantly between the groups.

The exclusion criteria ruled out a diagnosed comorbid substance use disorder other than heroin and nicotine; accordingly, the 26 patients (25.7%) recorded here reported recent, occasional use of another substance rather than meeting criteria for an additional substance use disorder, and this was more frequent during detoxification (42.9% vs. 9.6%). The specific substances involved, the time since last use and the amount/frequency of use were not systematically recorded. Smoking amount was comparable between the groups (Table 2; *χ*^2^(4) = 1.46, *p* = 0.834), with most patients smoking about one pack per day, although smoking duration and pack-years were not collected.

### 3.2 Clinical parameters

The two groups differed markedly on behavioral and affective measures (Table 4). Compared with patients in remission, those in detoxification had higher median scores for depression [6.00 (0–8) vs. 3.00 (0–8)], anxiety [7.00 (0–12) vs. 2.00 (0–11)], anger [3.00 (0–4) vs. 1.00 (0–4)], somatic symptoms [5.00 (0–8) vs. 2.00 (0–8)], suicidal ideation [2.00 (0–4) vs. 0.00 (0–4)], psychosis [2.00 (0–8) vs. 0.00 (0–8)], sleep problems [3.00 (0–4) vs. 1.00 (0–4)], dissociation [2.00 (0–4) vs. 0.00 (0–4)], and substance craving [19.00 (0–30) vs. 0.00 (0–12)] (all *p* ≤ 0.001). Conversely, healthy-lifestyle behavior (77.28 ± 18.03 vs. 59.71 ± 17.05) and healthy nutrition attitude (68.87 ± 10.67 vs. 58.22 ± 13.55) were significantly higher in the remission group (both *p* < 0.001).

**Table 4:** Comparative evaluation of psychometric measures and redox/ischemia biomarkers.

Variable	Remission (*N* = 52)	Detoxification (*N* = 49)	Test	*p*
Psychometric/symptom measures				
Depression	3.00 (0–8)	6.00 (0–8)	MWU = −5.34	<0.001
Anxiety	2.00 (0–11)	7.00 (0–12)	MWU = −5.11	<0.001
Anger	1.00 (0–4)	3.00 (0–4)	MWU = −5.33	<0.001
Mania	2.00 (0–6)	3.00 (0–8)	MWU = −1.58	0.114
Somatic symptoms	2.00 (0–8)	5.00 (0–8)	MWU = −4.13	<0.001
Suicidal ideation	0.00 (0–4)	2.00 (0–4)	MWU = −4.99	<0.001
Psychosis	0.00 (0–8)	2.00 (0–8)	MWU = −3.29	0.001
Sleep problems	1.00 (0–4)	3.00 (0–4)	MWU = −4.31	<0.001
Dissociation	0.00 (0–4)	2.00 (0–4)	MWU = −4.94	<0.001
Substance craving (total)	0.00 (0–12)	19.00 (0–30)	MWU = −7.77	<0.001
Healthy lifestyle behavior (total)	77.28 ± 18.03	59.71 ± 17.05	*t* = 5.02	<0.001
Healthy nutrition attitude (total)	68.87 ± 10.67	58.22 ± 13.55	*t* = 4.40	<0.001
Thiol-disulfide and IMA biomarkers				
Native thiol (μmol/L)	282.26 ± 41.85	270.43 ± 47.03	*t* = 1.34	0.184
Total thiol (μmol/L)	320.93 (217.05–418.40)	320.30 (148.20–428.55)	MWU = −0.74	0.459
Disulfide (μmol/L)	19.79 ± 7.23	20.02 ± 7.24	*t* = −0.16	0.870
Disulfide/Native thiol	7.13 ± 2.72	7.52 ± 2.68	*t* = −0.73	0.465
Disulfide/Total thiol	6.14 ± 2.07	6.45 ± 2.03	*t* = −0.74	0.459
Native thiol/Total thiol	87.71 ± 4.14	87.10 ± 4.06	*t* = 0.74	0.459
IMA (ABSU)	0.757 (0.020–1.183)	0.792 (0.030–1.164)	MWU = −1.50	0.134

Normally distributed continuous variables are presented as mean ± SD and non-normally distributed continuous variables as median (minimum-maximum). *t*, Student’s *t*-test; MWU, Mann–Whitney *U* test; IMA, ischemia-modified albumin; ABSU, absorbance units. *p* < 0.05 was considered statistically significant. Effect sizes (Cohen’s *d* or rank-biserial *r*) with 95% CI are reported in the text.

### 3.3 Thiol/disulfide and ischemia markers: group differences, correlates and predictors

In contrast to the psychometric measures, no significant differences were observed between treatment phases for any redox or ischemia biomarker (Table 4). Native thiol, total thiol, disulfide, the three derived ratios, and IMA were all comparable between groups (all *p* > 0.05). Median total thiol was 320.93 (217.05–418.40) μmol/L in the remission group and 320.30 (148.20–428.55) μmol/L in the detoxification group (*p* = 0.459). Median IMA was 0.757 (0.020–1.183) ABSU and 0.792 (0.030–1.164) ABSU, respectively (*p* = 0.134). Effect sizes for these comparisons were small (Cohen’s *d* 0.03–0.32). For IMA, the between-phase difference corresponded to Cohen’s *d* = −0.32 (95% CI − 0.72 to 0.07; mean difference −0.079 ABSU, 95% CI − 0.173 to 0.016); because this interval is wide and extends to a moderate effect, the non-significant IMA result should be regarded as inconclusive rather than as evidence of no difference, and the study was underpowered to detect small-to-moderate effects.

Medication-adjusted analyses yielded the same overall pattern (Table 5). After adjustment for medication use, treatment phase was not significantly associated with native thiol (*p* = 0.173), total thiol (*p* = 0.107), disulfide (*p* = 0.206), disulfide/native thiol (*p* = 0.468), disulfide/total thiol (*p* = 0.505), native thiol/total thiol (*p* = 0.505), or IMA (*p* = 0.145). Medication use itself was significantly associated only with disulfide levels [*F*(1, 98) = 4.049, *p* = 0.047], while its effects on the remaining biomarkers were not statistically significant.

**Table 5:** Medication-adjusted comparisons of redox and ischemia biomarkers between remission and detoxification groups.

Biomarker	Remission adjusted mean	Detoxification adjusted mean	Group *F*	Group *p*	Medication *F*	Medication *p*
Native thiol (μmol/L)	285.980	269.087	1.888	0.173	0.355	0.553
Total thiol (μmol/L)	329.577	307.686	2.651	0.107	1.283	0.260
Disulfide (μmol/L)	21.798	19.299	1.618	0.206	4.049	0.047*
Disulfide/native thiol	7.814	7.278	0.531	0.468	3.338	0.071
Disulfide/total thiol	6.642	6.268	0.447	0.505	3.062	0.083
Native thiol/total thiol	86.715	87.463	0.447	0.505	3.062	0.083
IMA (ABSU)	0.669	0.768	2.161	0.145	0.191	0.663

Estimated marginal means were obtained from general linear models including treatment group and medication use as main effects. The group-by-medication interaction was not included because of the highly unbalanced medication distribution between treatment groups. Group and medication effects are reported with *F*(1, 98) tests. *p < 0.05 was considered statistically significant.

These null findings were robust to adjustment. In multivariable models that included treatment phase together with medication use, recent non-heroin substance use, duration of heroin use and age at first treatment, treatment phase remained a non-significant predictor of both IMA (*B* = 0.092, *p* = 0.198) and total thiol (*B* = −20.15, *p* = 0.161). Likewise, when the 26 patients with recent non-heroin substance use were excluded (remission *n* = 47, detoxification *n* = 28), the groups still did not differ in IMA (*p* = 0.861), total thiol (*p* = 0.710) or all derived ratios (all *p* > 0.5). For the IMA comparison, the observed effect size (*d* = 0.32) corresponded to a statistical power of only 0.37 at *α* = 0.05; approximately 151 patients per group would be required to detect an effect of this magnitude with 80% power.

The biomarkers were associated with psychiatric symptoms and health behavior rather than with substance-use history (Tables 6, 1). Across the sample, none of the biomarkers correlated with age, BMI, duration of heroin use, daily amount, or time since last heroin use (Table 6). In contrast, several correlations were found with affective symptoms and health behaviors (Table 1). IMA correlated positively with depression (*r* = 0.254, *p* < 0.05) and craving (*r* = 0.209, *p* < 0.05). The disulfide/native thiol and disulfide/total thiol ratios correlated negatively with healthy-lifestyle (*r* = −0.237, *p* < 0.05) and nutrition attitude (*r* = −0.203, *p* < 0.05), whereas the native thiol/total thiol ratio showed correlations of similar magnitude in the opposite direction, indicating a shift toward the reduced (antioxidant) side of the redox balance in patients with healthier behaviors. None of these correlations remained significant after Bonferroni correction (*α* = 0.0014; smallest *p* = 0.010); they are therefore reported as exploratory, hypothesis-generating findings.

**Table 6:** Spearman correlations between biomarkers and demographic/clinical variables.

Variable	Age (years)	BMI (kg/m^2^)	Duration of heroin use (years)	Amount of heroin (g/day)	Time since last heroin use
Native thiol (μmol/L)	0.118	−0.066	0.082	−0.005	−0.083
Total thiol (μmol/L)	0.058	−0.078	0.033	0.066	−0.062
Disulfide (μmol/L)	−0.109	−0.010	−0.081	0.132	−0.088
Disulfide/native thiol	−0.173	0.041	−0.103	0.141	−0.068
Disulfide/total thiol	−0.173	0.041	−0.103	0.141	−0.068
Native thiol/total thiol	0.173	−0.041	0.103	−0.141	0.068
IMA (ABSU)	−0.032	−0.119	−0.092	0.067	0.004

Values are Spearman’s rank correlation coefficients (*r*).

Hierarchical multiple regression analyses are presented in Tables 7, 8. For total thiol, the baseline model including demographic and substance-use variables was not statistically significant (*R*^2^ = 0.035, adjusted *R*^2^ = −0.038, *F*(7, 93) = 0.479, *p* = 0.847). Adding depression, substance craving, and healthy lifestyle behavior in the second block explained an additional 1.9% of the variance; however, this increase was not statistically significant (Δ*R*^2^ = 0.019, Δ*F*(3, 90) = 0.608, *p* = 0.611). The final model remained non-significant (*R*^2^ = 0.054, adjusted *R*^2^ = −0.051, *F*(10, 90) = 0.514, *p* = 0.876), and none of the added variables independently predicted total thiol.

**Table 7:** Hierarchical multiple linear regression analysis of factors affecting total thiol.

Predictor	*B*	SE	*β*	*t*	*p*	VIF
Model 1						
Age	0.233	0.878	0.030	0.266	0.791	1.211
BMI	−1.570	1.203	−0.135	−1.305	0.195	1.025
Duration of heroin use	0.151	1.121	0.015	0.135	0.893	1.210
Daily heroin amount	2.018	2.917	0.075	0.692	0.491	1.132
Time since last heroin use	−0.053	0.501	−0.011	−0.105	0.917	1.037
Medication use	1.876	11.092	0.018	0.169	0.866	1.118
Alcohol use	10.318	10.583	0.104	0.975	0.332	1.107
Model 2						
Age	−0.035	0.920	−0.005	−0.038	0.969	1.314
BMI	−1.631	1.224	−0.140	−1.333	0.186	1.046
Duration of heroin use	0.420	1.158	0.042	0.363	0.718	1.276
Daily heroin amount	1.608	2.966	0.060	0.542	0.589	1.156
Time since last heroin use	−0.162	0.514	−0.033	−0.314	0.754	1.077
Medication use	−10.794	14.905	−0.105	−0.724	0.471	1.993
Alcohol use	11.350	10.797	0.115	1.051	0.296	1.138
Depression	0.790	2.491	0.039	0.317	0.752	1.473
Healthy lifestyle behavior	0.167	0.318	0.067	0.526	0.600	1.539
Substance craving	−0.890	0.792	−0.176	−1.124	0.264	2.321

Model	*R* ^2^	Adjusted *R*^2^	*F*	*p*	Δ*R*^2^	Δ*F*	Δ*p*
Model 1	0.035	−0.038	0.479	0.847	–	–	–
Model 2	0.054	−0.051	0.514	0.876	0.019	0.608	0.611

**Table 8:** Hierarchical multiple linear regression analysis of factors affecting ischemia-modified albumin (IMA).

Predictor	*B*	SE	*β*	*t*	*p*	VIF
Model 1						
Age	0.001	0.004	0.027	0.243	0.809	1.211
BMI	−0.006	0.006	−0.109	−1.056	0.294	1.025
Duration of heroin use	−0.002	0.006	−0.032	−0.283	0.778	1.210
Daily heroin amount	0.013	0.015	0.098	0.905	0.368	1.132
Time since last heroin use	−0.001	0.003	−0.025	−0.236	0.814	1.037
Medication use	−0.039	0.056	−0.076	−0.701	0.485	1.118
Alcohol use	0.016	0.053	0.033	0.306	0.760	1.107
Model 2						
Age	0.004	0.005	0.096	0.831	0.408	1.314
BMI	−0.006	0.006	−0.100	−0.974	0.333	1.046
Duration of heroin use	−0.004	0.006	−0.081	−0.708	0.481	1.276
Daily heroin amount	0.018	0.015	0.133	1.229	0.222	1.156
Time since last heroin use	0.000	0.003	0.015	0.143	0.887	1.077
Medication use	0.046	0.074	0.089	0.626	0.533	1.993
Alcohol use	−0.004	0.053	−0.008	−0.072	0.942	1.138
Depression	0.018	0.012	0.184	1.501	0.137	1.473
Healthy lifestyle behavior	0.000	0.002	0.039	0.314	0.754	1.539
Substance craving	0.005	0.004	0.202	1.313	0.192	2.321

Model	*R* ^2^	Adjusted *R*^2^	*F*	*p*	Δ*R*^2^	Δ*F*	Δ*p*
Model 1	0.028	−0.045	0.388	0.907	–	–	–
Model 2	0.084	−0.018	0.822	0.609	0.055	1.809	0.151

For IMA, the baseline model was also not statistically significant (*R*^2^ = 0.028, adjusted *R*^2^ = −0.045, *F*(7, 93) = 0.388, *p* = 0.907). Addition of depression, substance craving, and healthy lifestyle behavior increased the explained variance by 5.5%, but this improvement was not statistically significant (Δ*R*^2^ = 0.055, Δ*F*(3, 90) = 1.809, *p* = 0.151). The final model remained non-significant (*R*^2^ = 0.084, adjusted *R*^2^ = −0.018, *F*(10, 90) = 0.822, *p* = 0.609), and none of the added psychometric variables showed an independent predictive effect.

## 4 Discussion

In our study, we compared two clinical phases of heroin use disorder, sustained remission and acute detoxification, and examined how thiol-disulfide parameters and ischemia-modified albumin (IMA) relate to symptoms, craving, nutrition attitude, and healthy lifestyle. Our central finding was a clear separation between two kinds of measure. The behavioral and affective variables distinguished the two phases sharply, whereas the redox and ischemia markers did not. Instead, these markers were associated with symptom severity and health behavior across the sample as a whole. Within heroin use disorder, therefore, peripheral redox markers appear to function as continuous correlates of clinical state rather than as categorical indicators of treatment phase.

As expected, patients in the detoxification group exhibited higher self-reported symptom scores across multiple psychiatric screening domains compared with those in remission. Specifically, higher scores were observed in screening domains for depression, anxiety, anger, somatic symptoms, suicidal ideation, psychosis-like symptoms, sleep disturbance, and dissociation, alongside a markedly higher level of substance craving. This pattern is consistent with the acute psychological distress characteristic of the withdrawal phase and aligns with the well-documented role of emotional dysregulation in opioid dependence (Sloan et al., 2017; Jiang et al., 2022). Conversely, the remission group demonstrated higher scores on health-protective behavior scales, including overall healthy lifestyle and nutritional attitude measures. Together, these psychometric profiles illustrate two clinically distinct states within the same disorder, providing the behavioral context against which the biomarker findings should be evaluated.

Aside from the psychometric findings of the study, none of the thiol-disulfide or IMA parameters differed between the remission and detoxification groups. This result should be considered informative rather than merely negative. A companion case–control study from our group reported differences in thiol/disulfide-related ratios and IMA between patients with heroin use disorder and healthy controls (Yalcin Sahiner et al., 2025). When evaluating our findings together with our group’s previous case–control study (Yalcin Sahiner et al., 2025), several methodological and clinical differences should be underlined. Yalcin Sahiner et al. detected significant changes in thiol/disulfide ratios and IMA levels when comparing patients with heroin use disorder to healthy controls. Our study, on the other hand, focused solely on comparisons between the active detoxification and sustained remission stages within the course of the disorder. The absence of statistically significant differences between these two clinical stages suggests that redox imbalance and ischemia markers may remain altered during early-to-intermediate remission as well, potentially exhibiting a profile similar to that observed during active exposure. The differences between the two studies may stem from distinctions in sample characteristics and study design. The present finding suggests that, once the disorder is established, transitioning between acute withdrawal and stabilised remission does not, in itself, produce a measurable shift in systemic thiol chemistry. The contrast with bipolar disorder, where these same markers changed from acute mania to early remission (Korkmaz et al., 2023), suggests that the phase sensitivity of redox markers may be disorder-specific. More broadly, the direction of redox findings in addiction has been inconsistent across studies (Kotan et al., 2017; Kabadayı Şahin et al., 2023; Ergin Tuncay et al., 2023), which is consistent with markers that depend on clinical context rather than following a single, fixed trajectory. Maintenance buprenorphine/naloxone was far more frequent in the remission group (Table 3), so treatment phase and medication status are confounded in this design; the similarity of redox markers between the phases may partly reflect the antioxidant and neuroimmune effects of buprenorphine rather than a true biological equivalence.

As one of the important points of our study, although there was a marked difference between the two groups in terms of medication use, this pattern remained stable even after adjustments were made accordingly. Therefore, the absence of a difference between the two groups in terms of redox and ischemia biomarkers may not be fully explained solely by the higher frequency of medication use in the remission group. However, medication use showed a statistically significant relationship with disulfide levels, and for this reason, the findings cannot be interpreted as entirely independent of pharmacotherapy either. This relationship may indicate that pharmacological treatment could have a selective effect on redox homeostasis. The distribution of medication use between the two study groups is largely imbalanced, and our study’s findings have limited capacity to reflect differences in medication exposure. Therefore, these results should be interpreted with caution.

Moving on to the secondary marker, although IMA levels did not differ significantly between the two clinical phases, exploratory correlation analyses across the total sample revealed positive associations between IMA and both self-reported depressive symptom scores and substance craving severity. This aligns with previous literature reporting elevated IMA in mood and obsessive-compulsive spectrum domains, supporting the conceptualization of IMA as a composite marker of systemic oxidative, ischemic, and inflammatory stress rather than a single organ-specific mechanism (Karaaslan et al., 2019; Tunç et al., 2019; Shevtsova et al., 2021; Tampa et al., 2022). Because negative affective symptoms and craving levels peak during active withdrawal, the observed relationship between IMA and these psychometric indicators provides a plausible link between subjective distress and redox biochemistry, even in the absence of categorical group differences. The connection between redox status and craving is further supported by clinical trials demonstrating that the thiol-based antioxidant N-acetylcysteine reduces craving across various substance use disorders (Cuocina et al., 2024; Winterlind et al., 2024; Morley et al., 2023). This association should nonetheless be interpreted cautiously, it did not survive correction for multiple comparisons and the group-level difference in IMA, though non-significant, corresponded to a small-to-moderate effect that the study was underpowered to detect.

One prominent and internally consistent pattern linked the redox ratios to health behavior. Patients who reported healthier lifestyles and nutrition attitudes had lower disulfide/thiol ratios and a correspondingly higher native thiol/total thiol ratio, indicating a redox balance shifted toward the antioxidant side. Although a cross-sectional design cannot establish the direction of this association, the coherence of the correlations suggests that protective behavior and antioxidant reserve move together, and that lifestyle factors deserve attention when redox markers are interpreted in this population. These correlations did not survive Bonferroni correction and should be regarded as preliminary.

However, a notable finding of this study was the clear dissociation between clinical symptom burden and peripheral redox/ischemia markers. Patients in the detoxification phase exhibited significantly higher levels of depressive symptoms, anxiety, anger, somatic symptoms, suicidal ideation, psychotic symptoms, sleep problems, dissociation, and craving; in contrast, healthy lifestyle behaviors and healthy nutrition attitudes were more favorable among patients in remission. Despite these marked clinical and behavioral differences, none of the measured thiol-disulfide parameters or IMA showed a significant difference between the treatment phases. This suggests that changes in emotional and substance-related symptom severity across treatment phases are not necessarily accompanied by parallel changes in these peripheral biomarkers.

Multiple linear regression models did not identify any significant clinical predictor of total thiol or IMA, and the models explained little variance. These findings indicate that the determinants of these markers in heroin use disorder are not accounted for by age, body mass index, alcohol use, or simple parameters of heroin exposure. They may instead depend on a combination of symptoms, inflammation, nutrition, and pharmacotherapy (Butelman et al., 2023; Rowland et al., 2018), which would require larger samples and a wider set of covariates to model adequately.

This interpretation is further supported by our hierarchical regression analyses. After adjusting for demographic variables, substance use, and medication use, the addition of depression, substance craving, and healthy lifestyle behaviors to the model did not yield a significant improvement in predicting either total thiol or IMA levels. Although these psychometric variables demonstrated a clinically meaningful distinction between treatment groups, they failed to explain a significant proportion of the individual variability in either biomarker. Accordingly, our findings do not support the hypothesis that total thiol or IMA function as direct dimensional indicators of current depressive symptoms, substance craving, or health-related behaviors in patients with opioid dependence.

When interpreting the absence of statistically significant differences in thiol/disulfide homeostasis and IMA levels in our study, several confounding factors should be taken into account. The first of these is smoking. Active smoking was found to be nearly universal in both of our sample groups. Studies have shown that cigarette smoke significantly affects systemic redox balance and depletes circulating thiol pools (Zinellu et al., 2021). Interpreting this in the context of our study, this may have suppressed the subtle differences in endogenously generated oxidative stress between acute detoxification and remission, effectively masking potential phase-specific variance. Moreover, buprenorphine may be another factor that warrants consideration when interpreting oxidative and redox-related biomarkers in patients with heroin use disorder. Several studies conducted on heroin-dependent individuals receiving buprenorphine-naloxone treatment have shown that this treatment is characterized by decreased native and total thiol levels and increased disulfide formation, indicating alterations in thiol/disulfide balance (Kotan et al., 2017; Kabadayı Şahin et al., 2023). However, these studies show considerable limitations. In light of this information, the biochemical comparability observed between the two groups in our study may not reflect a genuine underlying physiological similarity. In order to isolate these two biological and pharmacological contributions, it will be important for future studies to account for smoking status and buprenorphine use.

Several limitations should be acknowledged. Since the design is cross-sectional, the associations we found cannot be interpreted as causal. The sample size is fairly small, which limited the power to detect small group differences and to build the regression models. Patients were grouped by their treatment phase at a single time point and were not followed over time. A few implausible height, BMI, and categorical values had to be corrected or treated as missing. Furthermore, self-report scales were used as the assessment instruments in this study, which may pose a limitation regarding the generalizability of the results. A key limitation of our study is the absence of a healthy control group. Our comparisons were conducted solely within a clinical sample consisting of patients with heroin use disorder. Thiol/disulfide and IMA levels in both groups could not be compared against reference values from a healthy population. In order to track the full course of the biochemical recovery process, prospective and longitudinal studies incorporating both healthy control groups and long-term abstinence follow-up are needed. Additionally, the study was conducted at a single center, and certain factors that could be considered important in terms of the variables included in the study were not controlled. The study included both men and women, but was not designed to compare between-sex parameters. Since redox physiology may differ by sex, further investigations could be made in this direction.

In our study, systemic inflammatory biomarkers were not directly measured. Therefore, statistical adjustment based on baseline inflammatory status could not be performed. Since smoking prevalence was 98.0% in both clinical groups, its independent contribution to oxidative stress and IMA levels could not be controlled for as a covariate in the regression models. Circulating albumin concentrations can directly influence IMA values. However, albumin levels were not assessed in our study, and this represents a methodological limitation. Nevertheless, both study groups had comparable BMI values (Table 2), and there were no clinical findings of severe liver disease or malnutrition. This reduces the likelihood of substantial albumin variance between the groups. For future studies, it is recommended that measurements of serum albumin levels be included. Finally, another methodological limitation of our study is the absence of correction for multiple statistical comparisons in the exploratory correlation and subgroup analyses. When multiple pairwise comparisons are performed simultaneously, this may lead to an increase in Type 1 error. This possibility cannot be disregarded, particularly for secondary outcomes with small effect sizes. Therefore, results that reach borderline statistical significance should be interpreted with caution. Furthermore, studies with larger sample sizes are needed to confirm these associations.

In conclusion, while acute detoxification and sustained remission in heroin use disorder are clearly distinguishable in terms of psychiatric symptoms, craving, and health behaviors, the redox and ischemia markers examined in this study did not provide a comparable distinction. These findings indicate that the assumption that treatment phase and symptom severity represent the same biological dimension does not hold true. Accordingly, total thiol and IMA, at least in the present sample, appear to have limited utility as standalone biomarkers for distinguishing detoxification from remission or for predicting current symptom burden. Future pre-registered, longitudinal, and sex-balanced studies incorporating healthy control groups are needed to more thoroughly track the complex biological recovery process in opioid dependence. Medication status and treatment phase were confounded and the correlational findings did not withstand correction for multiple comparisons, these conclusions are preliminary and require confirmation in larger, medication-controlled and longitudinal studies.

## Data Availability

The datasets used in this study were obtained from patients who provided informed consent, with their names and personal information kept strictly confidential. These datasets contain de-identified information. Due to ethical and legal restrictions, as well as the potential risk of re-identification of participants, the data cannot be publicly shared. However, upon reasonable request and subject to the approval of the corresponding author, de-identified analytical data may be made available, provided that appropriate institutional and ethical permissions are obtained.
